# MTGO-SC, A Tool to Explore Gene Modules in Single-Cell RNA Sequencing Data

**DOI:** 10.3389/fgene.2019.00953

**Published:** 2019-10-09

**Authors:** Nelson Nazzicari, Danila Vella, Claudia Coronnello, Dario Di Silvestre, Riccardo Bellazzi, Simone Marini

**Affiliations:** ^1^Research Centre for Fodder Crops and Dairy Productions, Council for Agricultural Research and Economics (CREA), Lodi, Italy; ^2^Bioengineering Unit, Ri. MED Foundation, Palermo, Italy; ^3^Istituti Clinici Scientifici Maugeri, Pavia, Italy; ^4^Computational Biology Unit, Ri. MED Foundation, Palermo, Italy; ^5^Institute of Biomedical Technologies, National Research Council, Segrate, Italy; ^6^Department of Electrical, Computer and Biomedical Engineering; Centre for Health, Technologies, University of Pavia, Pavia, Italy; ^7^Department of Surgery, University of Michigan, Ann Arbor, MI, United States

**Keywords:** single cell, RNA-seq, enrichment, gene network, clustering, gene module, annotation, scRNA-seq

## Abstract

The identification of functional modules in gene interaction networks is a key step in understanding biological processes. Network interpretation is essential for unveiling biological mechanisms, candidate biomarkers, or potential targets for drug discovery/repositioning. Plenty of biological module identification algorithms are available, although none is explicitly designed to perform the task on single-cell RNA sequencing (scRNA-seq) data. Here, we introduce MTGO-SC, an adaptation for scRNA-seq of our biological network module detection algorithm MTGO. MTGO-SC isolates gene functional modules by leveraging on both the network topological structure and the annotations characterizing the nodes (genes). These annotations are provided by an external source, such as databases and literature repositories (e.g., the Gene Ontology, Reactome). Thanks to the depth of single-cell data, it is possible to define one network for each cell cluster (typically, cell type or state) composing each sample, as opposed to traditional bulk RNA-seq, where the emerging gene network is averaged over the whole sample. MTGO-SC provides two complexity levels for interpretation: the gene-gene interaction and the intermodule interaction networks. MTGO-SC is versatile in letting the users define the rules to extract the gene network and integrated with the Seurat scRNA-seq analysis pipeline. MTGO-SC is available at https://github.com/ne1s0n/MTGOsc.

## Introduction

In contrast to bulk tissue RNA-sequencing, which allows mapping gene networks under the assumption of an “average” cell type, single-cell RNA sequencing (scRNA-seq) presents the unprecedented chance to study gene networks at the level of each cell type/state (usually identified by a cell cluster). In recent years, single-cell data repositories started to emerge, such as the Broad Institute Single Cell Portal^1^, scRNASeqDB^2^, and JingleBells^3^. Not surprisingly, a plethora of novel scRNA-seq computational tools have been recently published as well, addressing specific scRNA-seq data analysis challenges. To date, scRNA-tools^4^, a catalog of software packages designed for the analysis of scRNA-seq data, reports hundreds single-cell bioinformatics tools. These *in silico* methods are pipelines for scRNAseq downstream analysis, comprising quality control, imputation of drop-outs, batch effect correction, normalization, scaling, highly variable gene detection, clustering, and cluster characterization.

Interestingly, an unexplored niche in scRNAseq data analysis is gene network interpretation, i.e., grouping genes in functional modules to understand signal origins and cell functional organization. Gene modules are labeled with specific cellular function(s), such as metabolic pathways or signal transduction systems, and provide a picture of how the cellular machinery is organized. Gene network interpretation greatly benefits from computational tools, as hundreds to thousands of genes cannot be grouped without an automated approach. The interpretation of gene networks is a key step toward applications such as omics data integration, protein/gene function discovery, molecular mechanism comprehension, and drug target discovery/repositioning. In contrast with bulk RNA-seq, scRNA-seq data unlock the possibility to understand the different heterogeneous gene subnetworks underlying not a tissue as a whole but each cell community within that specific tissue. For example, in a tissue sample comprising different types of neural cells, it would be possible to isolate several gene networks corresponding to different types of neural cells, each one with its specific functional modules. This is particularly important if uncommon cell types are present since infrequent signals could be diluted and impossible to catch with bulk RNA-seq analysis. This motivates the need for a tool to find gene functional modules specifically designed for scRNA-seq data. This tool would analyze gene networks and, more importantly, would extract gene modules, specific for cell type/cell state, i.e., it would unveil a deeper level of mapping if, compared to bulk RNA-seq, gene networks built on the cell signal averaged over all the cells of the tissue sample.

**Figure 1:**
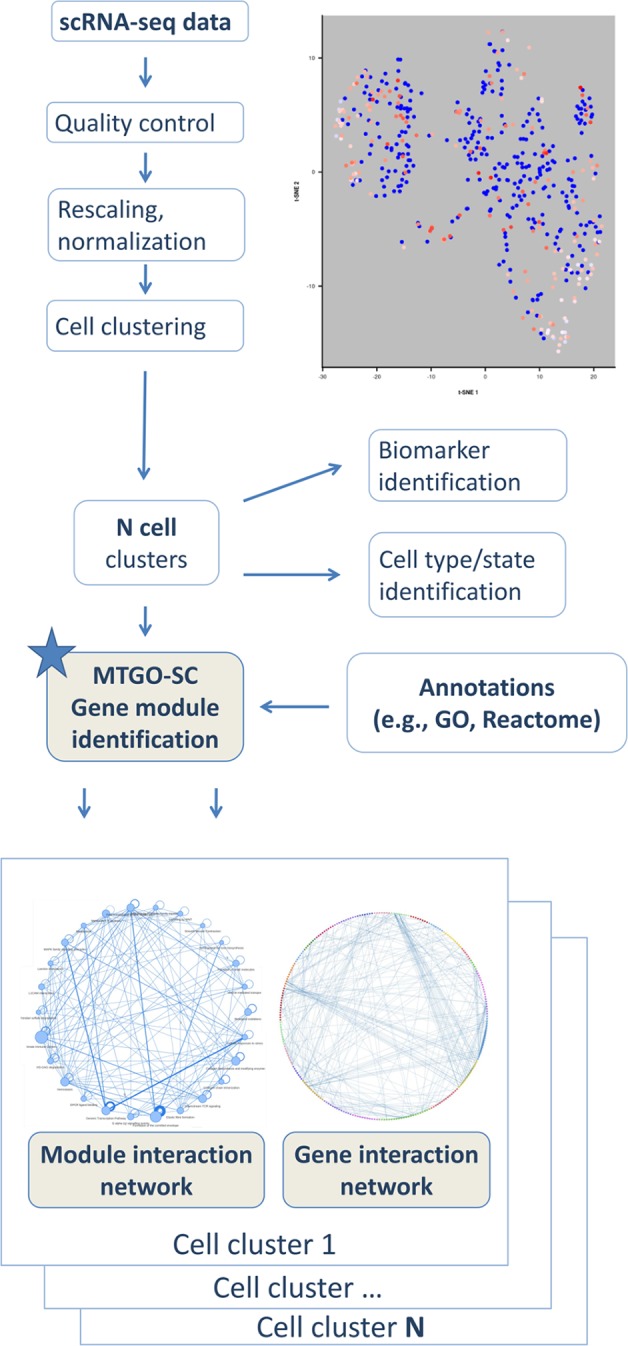
Schema of MTGO-SC. scRNA-seq data are analyzed through the typical pipeline including quality control, rescaling, normalization, and clustering. MTGO-SC provides a further postprocessing step, in which a cell cluster is analyzed to extract the gene network and by integrating it with an annotation source, such as the GO or Reactome, each gene network is parsed into modules describing the cell machinery.

To fulfill this task, here, we present MTGO-SC (MTGO for single-cell RNA seq). The algorithm is based on MTGO, presented by Vella et al. (2018), a novel system for biological network interpretation recently published by our group, which outperforms state-of-the art algorithms on module detection in protein networks. In particular, MTGO outperforms existing methods on different benchmark data sets and, more importantly, sharply increases the detection of sparse/small modules, which are typical in biological networks. MTGO-SC is a tool to perform module identification of gene networks tailored for scRNAseq, and it is intended to provide an additional postprocessing step in the scRNA-seq pipeline analysis (**Figure 1**). MTGO-SC is designed to produce an interpretable gene interaction network for each cell cluster resulting from scRNA-seq analysis. MTGO-SC allows the users to navigate the network on two interlaced levels, i.e., the gene-gene interaction and the module-module interaction. The gene-gene interaction network is the traditional gene network view, where each node is a gene and each edge is an interaction (for example, a gene expression correlation). Genes are grouped by MTGO-SC into functional modules characterized by a representative label (e.g., a GO term or a pathway), therefore greatly easing network interpretability. This is fundamentally different from a typical gene enrichment result, which provides only some statistically significant terms related to the whole network (as a group of genes to enrich). From the gene-gene interaction network, it is possible to extract the module-module interaction network, i.e., a network where each node is a labeled functional module (a group of genes). In summary, MTGO-SC provides both labeling terms and relational networks to study how genes and modules are interacting and relating to each other (**Figure 1**), thus opening a window over the cell machinery. MTGO-SC is also natively integrated with Seurat, a popular tool for scRNAseq downstream analysis by Butler et al. (2018).

## Materials and Methods

MTGO-SC is designed as postprocessing step of single-cell data analysis pipelines (**Figure 1**). Once the cells in a data set have been clustered, for example, by the analysis tool Seurat Butler et al. (2018), MTGO-SC extracts for each cell cluster a gene interaction network and identifies its gene modules. MTGO-SC pipeline has four steps: creation of the gene expression correlation matrix, network thinning, invocation of MTGO, and visualization of resulting network(s). MTGO-SC is an algorithm intended to extract functional modules from single-cell gene networks, not to clusters cells.

### Gene Expression Matrix

Given a gene expression matrix *E* = [*e* *_ij_*], with *e* *_ij_* *≥0* representing the expression of gene *i* in cell *j*, we define the symmetric correlation expression matrix *C* = [*c* *_ik_*] through the selection of a commutative metric function *f* (*x* *_1_* *,x* *_2_*), so that *c* *_ik_* = c*_ki_* = *f* (E*_i_*, E*_k_*), with *E* *_i_* and *E* *_k_* being two full rows of expression matrix *E*. The selected function must measure the expression similarity of two genes through the full set of the analyzed cell cluster. In addition, it must be robust to noise and minimize the impact of dropouts and data sparseness, two well-known problem in scRNA-seq, as discussed by Haque et al. (2017).

### Network Extraction

Gene expression correlation matrix *C* can formally be considered as the matrix representation of a weighted gene network, with each element *C* *_ik_* representing the weight on the edge connecting gene *i* to gene *k*. The described network is always fully connected, with all genes having an edge with all other genes. Moreover, the weights distribution is expected to be highly skewed, with the majority of genes showing little to no correlation and only a limited subset showing high (positive or negative) correlation. For this reason, we implemented a network extraction step to extract the meaningful subnetwork and remove uninformative connections. This step comprises two parts, namely, (a) calculation of the coexpression matrix and (b) thinning. A parameter combination to run MTGO-SC therefore depends on (a) the correlation method and (b) the thinning method. To calculate the expression network, the users can choose among Spearman, Person, Kendall, *p*, and ϕ correlations Skinnider et al. (2019). To thin the resulting network, the users can proceed by either fitting the network by selecting the top percentile coexpression (MTGO-SC considers top 10, 20, 30, 40, and 50 percentiles) or pruning the network to fit a scale-free law. A scale-free network is a network whose degree distribution follows a power law, typically with γ ∈ [2,3] Choromański et al. (2013). Biology is rich of scale-free network examples, including metabolic, protein, and gene networks Khanin and Wit (2006). In the case of gene networks, the scale-free principle is well integrated in modeling their evolution Zhang et al. (2015). We therefore designed a network thinning function that extracts an optimally scale-free subnetwork from the fully connected network. MTGO-SC thinning function implements a grid search over a set of cutoff values, i.e., a set of thresholds that edge weights must surpass in absolute value. For each cutoff value, edges (correlations) smaller in absolute value than the considered cutoff are removed from the network. It is then measured the goodness of fit on a power law of the obtained subnetwork. The cutoff value resulting in the best fit is selected and the resulting subnetwork is prepared for further elaboration.

A summary of the user-defined parameters for network extraction and MTGO processing v reported in **Table 1**. Once the networks are extracted from the subsection of the gene expression matrix corresponding to each cell cluster, they are individually processed with MTGO to infer their functional modules.

**Table 1:** MTGO-SC main parameters.

Function name	Function description	Parameters
write.coexpressionMatrix	Compute and save the correlation matrix	Location, overwriting options, gene interaction metric function (defaults to cor)
write.edges	Thins the network and saves the result	Location, overwriting options, thinning function (defaults to thinning_abs_threshold)
write.dictionary	Create and save a gene-term dictionary file	Location, the dictionary tuplets
export.network.modules	Save visual representation of functional modules	Module collapse toggle
thinning_abs_threshold	Subset a coexpression network for thresholded absolute values	The threshold value
thinning_percentile	Subset a coexpression network to the desired percentile	The percentile value
thinning_scale_free	Subset a coexpression network to maximise free scale fit	The target gamma, plus a grid of thresholds to be compared
coexpr_propr	Compute gene coexpression *via* proportionality functions	The selected function, plus any extra parameters
call.MTGO	Invoke the MTGO execution	Location containing all the data and config file

#### Assessing Extracted Networks

To help the users to choose the best parameter combination for gene network extraction (i.e., coexpression and thinning methods), we prepared a function to benchmark the extracted networks against a ground-truth network (GTN). The GTN is an experimentally confirmed collection of biological complexes. The level of overlapping between the modules found by MTGO-SC and the modules in the GTN is used as index of reliability of the proposed method in the form of a p-value derived from the affinity score (AS). The AS is defined as the squared number of homologous edges in the two networks divided by the multiplication of the number of edges for each network, as follows:

(1) $$AS(N,GTN)=\frac{h{\left(N,GTN\right)}^{2}}{N_{EN}^{}*N_{EGTN}^{}}$$

**Figure 2:**
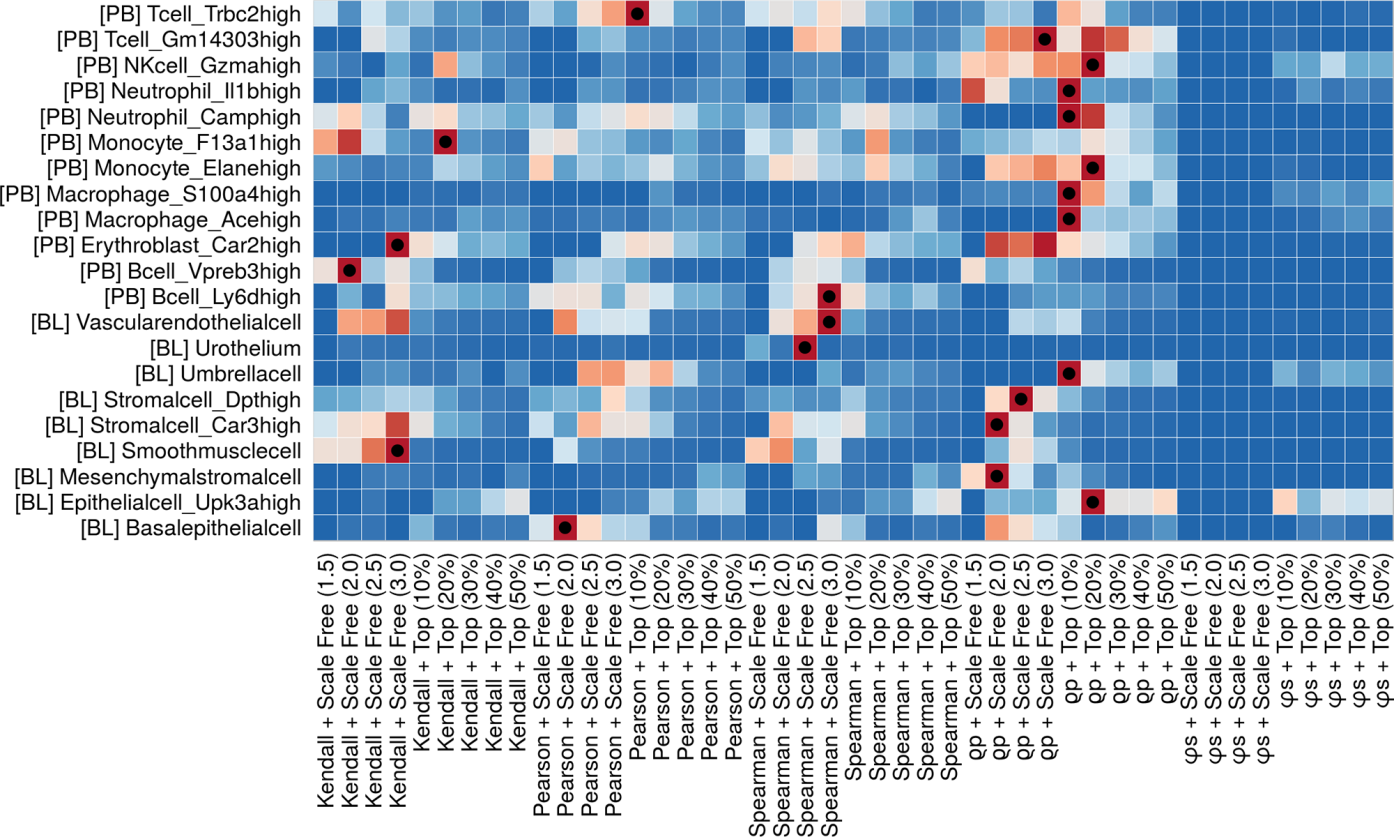
Ranking of all the network extraction approaches (coexpression metric combined with thinning technique) applied to the different cell types, based on affinity score (AS). The heatmap colors depend on the per-row rescaled −*log* (*P*-*value*) (blue to red). The best method per cell type is marked with a dot. Although some methods seem to fit many cell types a clear cut, one-size-fit-all solution does not emerge. The users can therefore tailor the network extraction method to the different cell types in his/her data.

Here, N is the extracted gene network with a specific parameter combination; GTN is the ground-truth network; *h*(*N*,*GTN*) is the number of homologous edges of N and GTN; *N* *_EN_* is the number of edges of N; *N* *_EGTN_* is the number of edges of GTN. After calculating the AS, we generate a population of equivalent random networks by rewiring the edges of N while preserving the original degree distribution. By comparing the AS with the rewired networks, we obtain a p-value associated to N. In this way, users can rank all the possible extracted networks for each cell type of their data, with the top scoring ones producing networks closest to the selected GTN. These results are visualized using a −*log*(*p*−*value* *_N_*) heatmap to find the best parameter combination for the different cell type (**Figure 2**). Different cell types (i.e., different heatmap rows) show different performances (i.e., colors in heatmap cells) over the methods. A dot marks the best performing ones. A detailed example of how to run MTGO-SC to find the best extraction method for each cell type is reported in the MTGO-SC package vignette.

While users can define their own GTN, the default one in MTGO-SC collects complexes derived from four different sources to maximize the experimental knowledge coverage used to evaluate the method. The four collections come from CORUM database Giurgiu et al. (2018), a protein interaction map Ramani et al. (2008), and String and Reactome databases Skinnider et al. (2019). The GTN involves 5,727 nodes and 67,756 edges. It has to be noted that our experimental knowledge on the biological complexes is still limited, and a GTN necessarily covers only partially and unevenly all the gene connections involved in a living organism. This problem is discussed in Section 3, along with suggestions to mitigate it.

### MTGO Principles

As MTGO-SC applies the MTGO algorithm for module identification, here, we report a summary of how MTGO works. MTGO is an algorithm for module identification in biological networks Vella et al. (2018). It is designed to consider two key aspects, i.e., the topological properties of the network (the connection arrangement) and the *a priori* knowledge about the biomolecules involved, represented by literature annotations, such as the Gene Ontology (GO).

With the goal of identifying relevant modules, MTGO iteratively builds network partitions, improving the quality at each iteration until a steady state is reached. A partition is a subdivision of the graph (i.e., the gene interaction network) into subgraphs, covering all the network nodes. MTGO generates at each step a new partition by mixing the nodes among the clusters. The result consists of both a network partition, thus a set of clusters, and a set of functional modules, representing the biological entities (pathway, complex, process, and component) associated to each cluster. The modules are reshaped taking into account two factors, i.e., the biological annotations and the graph topology. At the end of each iteration, MTGO measures the modularity (*Q*) Newman and Girvan (2004) and Quality GO (*QGO*) Vella et al. (2018) reached. *Q* represents the global quality of the partition in terms of modularity and is a state-of-the-art function aiming to evaluate graph partitions Fortunato (2010). Conversely, *QGO* evaluates the agreement between *C* and Φ, where *C* is the cluster partition, and Φ is the subset of the annotation source terms selected by MTGO to describe the biological functions linked to the partition *C* of the network. The ideal solution would have *C* and Φ overlapping, i.e., the algorithm should find the optimal modularity and optimal fitting for the annotation source constraints.

(2) $$Q(C^{k})={\sum_{1<h<H_{k}^{}}\frac{e_{h}^{k}}{\left|E\right|}-\left(\frac{d_{h}^{k}}{2*|E|}\right)}^{2}$$

*C* *^k^* is the partition for the iteration *k*; *H* *^k^* is the number of topological modules of the partition *k*; $e_{h}^{k}$ is the total number of edges in the *h*th topological module; $d_{h}^{k}$ is the sum of the node degrees of the *h*th topological module. *E* is the set of all the relationships between network node pairs. We define it as *E* *=* {*e*i,j | 1 < *i*,*j* < *N*, *I* ≠ *j*}. *Q* ranges from −1 to 1. A positive value implies that there are more links within topological modules than expected at random.

(3) $$QGO(C^{k})=\frac{\sum_{1<h<H_{k}^{}}|\delta_{B,h}^{k}\cap c_{h}^{k}}{N_{AnnSo}^{}}$$

Here, *B*,*h* *^k^* is an annotation term for the topological module *h* at the iteration *k*, whereas $c_{h}^{k}$) is the functional module minimizing the *Selection* γ function for the topological module $\delta_{B,h}^{k}$. The aim of *Selection* γ function is to choose an annotation term as model to drive the building process of a topological module. *N* *_AnnSo_* is the total number of nodes with at least one δ*_p_* assigned. δ*_p_* is set of network genes associated to a *p* annotation term.

We terminate the algorithm when two consecutive iterations show a negligible variation of *Q* and *QGO*, specifically when |*Q* *_k_*_+1_ – *Q* *_k_* < *T* | and *QGO* *_k_* – *QGO* *_k – 1_* < *T* |, with threshold *T* *=* 10−4.

A detailed description of the functioning of MTGO, including the pseudocode, is provided by Vella et al. (2018). An MTGO user manual is provided in its repository^5^.

### Visualization of Functional Modules

**Figure 3:**
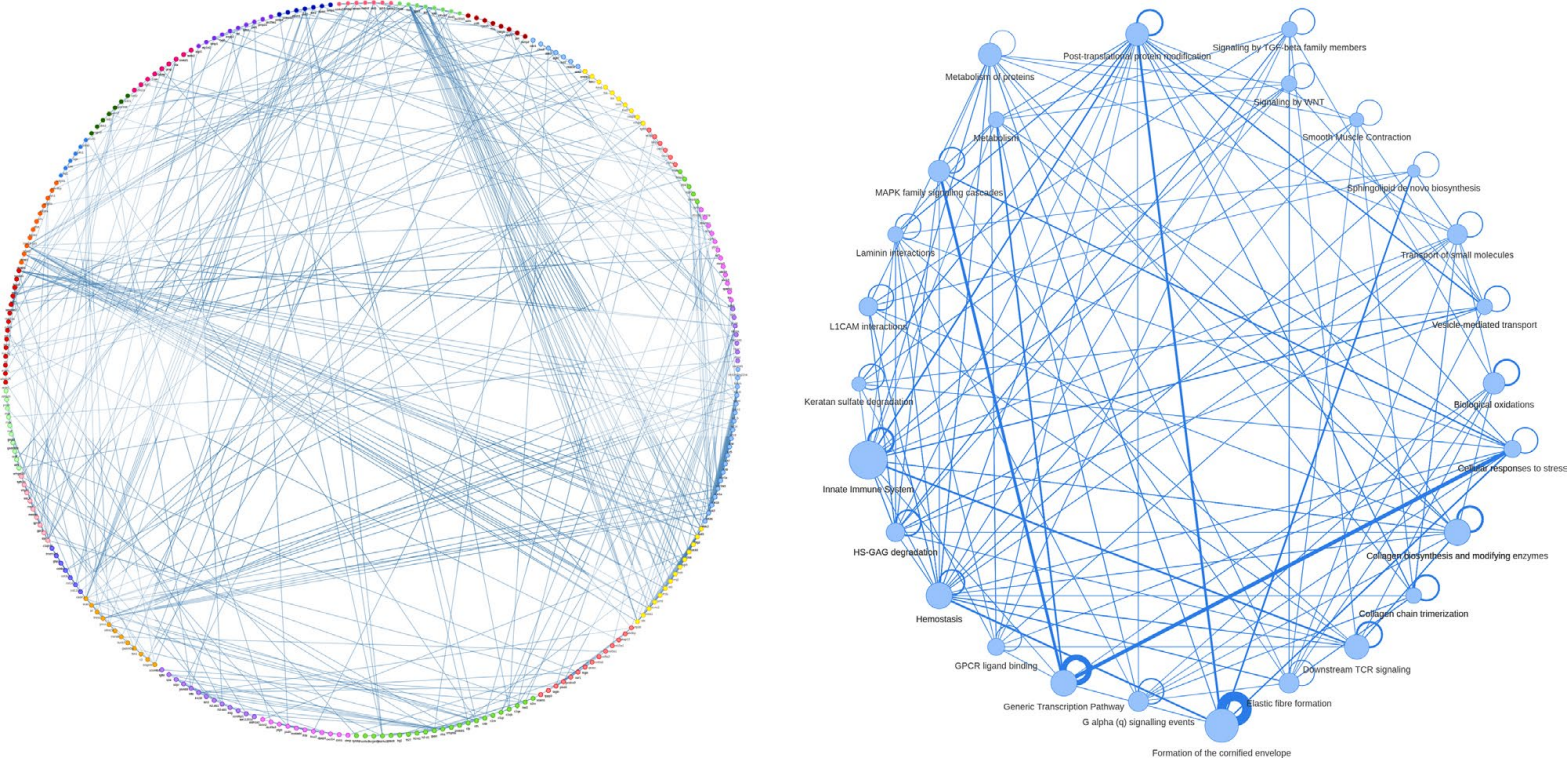
An example of gene network, extracted from basal epithelial cells of mouse bladder scRNA-seq. The whole gene network **(A)** is visualized with nodes colored by gene module (i.e., the annotation labels attributed by MTGO-SC to gene groups). The gene module network **(B)**, with each node representing a module extracted by MTGO-SC, has the gene belonging to the same functional module sharing the same color. The edge thicknesses reflect node correlation. The module network edges show self-loops representing the interactions of the genes *within* the modules. The thickness of these self-loops reflects the level of within-module correlation.

MTGO-SC produces a partition of the genes in a set of functional modules. MTGO-SC uses the visNetwork R package^6^ to produce a customizable graphical representation of MTGO numeric results. At the highest level, MTGO-SC can show the connection between functional modules, with the weight of the connection represented as edge thickness and the list of genes for each module reported *via* tooltip (**Figure 3B**). It is also possible to not collapse the genes into modules and produce the full network of examined genes (**Figure 3A**). In this case, modules are color-coded and reported in each gene’s tooltip. Finally, it is possible to isolate separated networks, one per module, for finer examination.

## Results and Discussion

MTGO-SC is a tool to infer gene-gene networks in scRNA-seq. MTGO-SC provides a further layer to scRNA-seq analysis—the study of gene-gene interactions—by isolating the different gene modules and labeling them with a specific enrichment term. MTGO-SC analysis grants flexibility as the users controls key parameters to define the gene network. In particular, how to select the nodes (genes), the method to calculate the gene coexpression network, including a function to benchmark all combinations of methods an parameters over a selected GTN (**Figure 2**), and one to compare the different methods over each single-cell type (**Supplemental Figure 1**); how to calculate gene interactions; and how the (weighted) edges of the network are defined, which annotation source is used for labeling (as default, we provide the GO and Reactome pathways, but users can use their own).

The clustering coherence for two extracted networks is the number of genes that ends up in the same cluster at the end of the processing divided by the average number of genes in the two extracted networks. This can be collected in an *N* × *N* square matrix (graphically, in a heatmap) where *N* is the total number of methods tried (i.e., all coexpression metrics × all network extraction algorithms). If rows and columns of the heatmap are sorted by AS, a good result will show a “hot spot” in the top right corner, meaning that top ranking methods show higher coherence to each other, and lower ranking methods are more scattered. An example of this comparison is provided in **Supplemental Figure 1**, showing the extraction method coherence for bladder smooth muscle cells.

To extract gene modules, MTGO-SC leverages not only the topological properties of the network but also the previous biological knowledge provided by the users. This knowledge comes in two forms, namely, (a) a GTN to find the best parameter combination for the gene network extraction and (b) an annotation source to partition the previously extracted gene network into functional modules. The drawback of using previous knowledge is that all sources are inherently biased. For example, the sources we used to build the GTN come from gene and protein databases, which in turn are usually not derived from the granularity of single-cell experiments. A gene network measured on the tissue level implies the assumption of an *average* cell-per-tissue type, therefore not considering the heterogeneity of cells unveiled in detail by single-cell analysis. Even if the source is diligently measured on the cell-type level, more studied cell types might weight more than less studied ones in the gene network definition. Finally, even the knowledge of single gene and protein interactions is unbalanced toward “superstar” genes or proteins, which are more studied than others Lam et al. (2016); Marini et al. (2018). Users should select the gene label annotation source carefully and according to the problem they want to analyze. For example, if the studied cells are affected by a specific condition, the source should ideally reflect that (i.e., it should ideally derive from, or at least include, cells from samples marked by that condition). MTGO-SC parameters can be tuned to curb their influences to mitigate the influence of this implicit source bias in both GTN and annotation source. First, in the network extraction phase, if users cannot find a GTN fitting the purpose of their study, they can focus the thinning to best fit the power law. Not only this makes sense biologically but from our empirical assessment (**Figure 2**), fitting the power law seems to provide best or high results in the largest part of the analyzed cell types. Secondly, in the module discovery phase, the influence of the annotation source can be greatly limited by the selection of MTGO partition which maximizes the mean cluster density, therefore weighting more the adherence of the discovered modules to the network structure (topology) and less on the overlapping between discovered modules and annotation source [this topic is treated in detail in the MTGO paper of Vella et al. (2018)]. In this way, users can reduce the influence (and therefore the bias) introduced by the GTN and annotation source.

### Gene Modules Out of the Mouse Gene Atlas and Reactome

To test our pipeline, we applied it to the bladder and peripheral blood mouse tissue provided by batch-removed MCA data sets of the corresponding tissues, described by Han et al. (2018). We obtained 2,297 and 2,356 cells for bladder and peripheral blood, respectively, with an MCA cell-type label. We then filtered the said cell clusters for minimum number of genes expressed per cell (500), maximum UMIs per cell (10,000), and maximum fraction of expressed mitochondrial genes per cell (0.1), obtaining two reduced sets of 2,297 and 2,117 cells for bladder and peripheral blood, respectively. Finally, we discarded the cell clusters with less than 50 cells. Our retained cell clusters were characterized in bladder as stromal cells (two clusters, Dpt high or Car3), vascular endothelial cells, urothelium cells, smooth muscle cell, basal epithelial cells, epithelial cell (Upk3a high), mesenchymal stromal cells, and umbrella cells; and in peripheral blood as B cells (two clusters, Ly6d or Vpreb3 high), erythroblasts, macrophages (two clusters, Ace or S100a4 high), monocytes (two clusters, Elane or F13a1 high), neutrophils (two clusters, Camp or Il1b high), NK cells, T cell (two clusters, Gm14303 or Trbc2 high).

We selected the highly variable genes according to the Seurat pipeline (FindVariableGenes, mean.function = ExpMean, dispersion.function = LogVMR, x.low.cutoff = 0.5, x.high.cutoff = 8, y.cutoff = 0.5, num.bin = 20). Gene networks were computed with a grid search along all the parameter combinations (coexpression and thinning methods) and selecting the best ones according to our assessment method based on the AS 2.2.1. Characteristics of the cell types and of the obtained networks are reported in **Supplemental Tables 1** and **2**.

After the application of MTGO-SC, each cell cluster is characterized by a gene network. In each gene network, genes are clustered in modules. Each gene module is labeled by a Reactome pathway characterizing the cellular machinery in which the group of genes is likely involved. Unlike traditional enrichment, MTGO-SC infers also the gene module interactions (**Figure 3**).

#### Gene Networks of the Bladder Cell Types

Almost all the cell types constituting the mouse bladder in MCA (7/9) showed the Formation of the Cornified envelope as enriched pathway. The cornified envelope is a protein structure with a scaffold function for lipid attachment, typical of epidermal regions Candi et al. (2005). In fact, the protein precursor of the epidermal cornified envelope is the Involucrin, also detected in the differentiating epithelial cells of normal tongue, oesophagus, and bladder Li et al. (2000).

Excluding generic terms related to metabolism, muscle contraction (4/9; epithelial cells Upk3a high, smooth muscle cells, umbrella cells, vascular endothelial cells), Molecules associated with elastic fibres (3/9; smooth muscle cells, stromal cells Dpt high, umbrella cells) and collagen biosynthesis and modifying enzymes (3/9; epithelial cells Upk3a high, smooth muscle cells, umbrella cells) resulted common highlighting the muscular and elastic nature of the bladder Chunhui et al. (2014).

Concerning pathways characterizing specific cell types, it is interesting the presence of “Post-translational modification: synthesis of GPI-anchored proteins” in epithelial cells Upk3a high and vascular endothelial cells. In fact, glycoproteins constitute the glycosaminoglycan (GAG) layer which has a protective role in the bladder providing a barrier against the penetration of toxic agents, urine, and bacteria Klinger (2016). In addition, Upk3a, highly expressed in in Epithelial cells Upk3a high, contributes to the formation of urothelial glycocalyx, which may play an important role in preventing bacterial adherence, as well as toll-like receptor (TLR) family (urothelium bladder and vascular endothelial cells) which have a role in urinary tract defense against pathogenic microbial agents Behzadi and Behzadi (2016).

Of note, it is very interesting that the signaling by VEGF, collagen chain trimerization, and Heparan sulfate/heparin (HS-GAG) metabolism was specifically enriched in vascular endothelial cells. In fact, it well established the relation of angiogenesis with VEGF Coultas et al. (2005), collagen Feng et al. (2013), and heparan sulfate/heparin Chiodelli et al. (2015).

#### Gene Networks of the Peripheral Blood Cell Types

As for peripheral blood it is not surprising that, due to the nature of cell type selected, the most shared pathway concern immune system (neutrophil degranulation, immunoregulatory interactions between a lymphoid and a nonlymphoid cell, metal sequestration by antimicrobial proteins signaling by Interleukins, MHC class II antigen presentation).

In addition, mitotic spindle checkpoint and G2/M transition, selected for macrophage S100a4 high, were in agreement with the high expression of S100a4 involved in the regulation of a number of cellular processes such as cell cycle progression and differentiation Orre et al. (2013).

Similarly, different pathways related to cell cycle (mitotic prophase, amplification of signal from the kinetochores, S phase, SUMOylation of DNA replication proteins, kinesins) were specifically enriched in Bcell Vpreb3 high.

Of note, different pathways related to immune system (TNFR2 noncanonical NF-kB pathway, activation of matrix metalloproteinases, DAP12 signaling) were specifically enriched in Tcell Gm14303 high. Different studies have described the relation between TNRF2 and T lymphocyte Salomon et al. (2018), between DAP12 and T lymphocyte Haura et al. (2015); Salomon et al. (2018), as well as between the activation of matrix metalloproteinases and T lymphocyte Edsparr et al. (2011); Hayes et al. (2014).

Again, the specific enrichment of Senescence-Associated Secretory Phenotype (SASP) in NK cell Gzma high is interesting. In fact, their relation has been recently described Ruscetti et al. (2018); Antonangeli et al. (2019).

### Literature Search of MTGO-SC Pairs of Cell Type and Terms

As a further step in our analysis, we performed an automated literature search of the Reactome pathways attached by MTGO-SC to the gene modules and their related cell type. Specifically, we tested whether the pathways obtained with MTGO-SC, and the pathways enriched with over-expressed genes, were consistent with existing published results. We therefore performed an automated search on the Pubmed repository by using RISmed^7^, reported in **Supplemental Figures 2** and **3** for bladder and peripheral blood, respectively. For each cell type, we considered two sets of pathways, those revealed by MTGO-SC and those obtained by enrichment analysis, performed with ReactomePA Yu and He (2016). Since the enrichment provides a large number of pathways, each cell type is selected a subset with a size equal to the set of pathways extracted by MTGO-SC, taking the pathways with the lowest values of adjusted p-value. For the analyzed tissues, blood, and bladder, we considered all the possible couples pathways-cell type. For each obtained combination, we counted the number of papers that a Pubmed repository search (from 2000 to 2019) detects by looking the corresponding string. We used the hypergeometric distribution to assess whether, among the papers concerning the selected tissue, the number of hits obtained by searching a combination of pathway and cell type is significantly higher than what expected by chance. A combination is considered significantly consistent with published results if the p-value based on the hypergeometric distribution is lower than 0.05. In order to obtain more hits in Pubmed search, when the cell-type name refers to a highly expressed genes, for instance, “Neutrophil Il1b high”, we implemented the Pubmed search also after splitting the cell-type original string in the two component, i.e., the general cell type (Neutrophil) and the specific genes (Il1b). For example, in **Supplemental Figure 3**, “Neutrophil” and “Il1b” are also included in the hits matrix. Here, only the combinations of cell types (splitted or not) and the pathway that bring to a significant number of Pubmed search hits are shown. More detailed information about the Pubmed search hits and the obtained p-values is included in the **Supplemental Table 1**. Results show that the meaningful and different statistically significant pair of terms are retrieved by both MTGO-SC and ReactomePA. This outcome suggests that the two methods are complementary and should be combined. We therefore integrated the ReactomePA + RISmed literature search in the MTGO-SC pipeline.

## Conclusions

MTGO-SC (MTGO for single-cell RNA-seq) is a novel pipeline for single-cell biological network interpretation. Specifically, MTGO-SC requires as input the digital gene expression matrix of scRNA-seq data, and an annotation source of gene labels, such as the GO. The output is the partition of the network in gene modules. Each of these modules is associated to a specific label present in the gene label source. MTGO-SC is intended to be used as an additional step of data interpretation in the typical scRNA-seq pipeline and, in particular, to be applied separately to each cell cluster previously emerged. Furthermore, MTGO-SC provides also a gene network extraction assessment function that allows the users to compare the networks extracted with different coexpression calculation methods and thinning parameters to tailor the extraction methods to the different cell types. MTGO-SC is presented as natively integrated with Seurat, a popular R tool for scRNA-seq analysis. MTGO-SC comes with customizable parameters to extract the gene network and to find the gene modules. To ease the network interpretation, MTGO-SC provides both gene and functional module networks, as well as literature enrichment.

## Data Availability Statement

The data sets and code for this study can be found at https://github.com/ne1s0n/MTGOsc. MTGO-SC is also available as an R package at https://rdrr.io/github/ne1s0n/MTGOsc/.

## Author Contributions

SM, NN, and DV conceived MTGO-SC and the experiments and prepared the data; NN, DV, CC, and SM wrote the code and performed the experiments; SM, CC, and DS analyzed the data; SM, DV, and NN wrote the paper; RB, SM, DV, NN, DS, and CC revised the paper.

## Funding

Fondazione Regionale Ricerca Biomedica - Genomic profiling of rare hematologic malignancies, development of personalized medicine strategies, and their implementation into the Rete Ematologica Lombarda (REL) clinical network.

## Conflict of Interest

The authors declare that the research was conducted in the absence of any commercial or financial relationships that could be construed as a potential conflict of interest.

## Footnotes

1
https://portals.broadinstitute.org/single_cell

2
https://bioinfo.uth.edu/scrnaseqdb

3
http://jinglebells.bgu.ac.il

4
http://www.scrna-tools.org/

5
https://gitlab.com/d1vella/MTGO

6
https://github.com/datastorm-open/visNetwork

7
https://CRAN.R-project.org/package=RISmed

## Supplementary Material

Figure 1 Comparison of different methods to extract the gene network from smooth muscle cell coomunity in bladder.

Figure 2 Rismed search on the Pubmed repository for bladder terms.

Figure 3 Rismed search on the Pubmed repository for peripherl blood terms.

Table 1 Characteristics of the cell communties.

Table 2 Characteristics of the networks extracted from the cell communties.
