# Supplementary figures and images for: MTGO-SC, A Tool to Explore Gene Modules in Single-Cell RNA Sequencing Data
Source: Front Genet. 2019 Oct 9;10:953. doi: 10.3389/fgene.2019.00953 (PMC6794379; doi:10.3389/fgene.2019.00953)

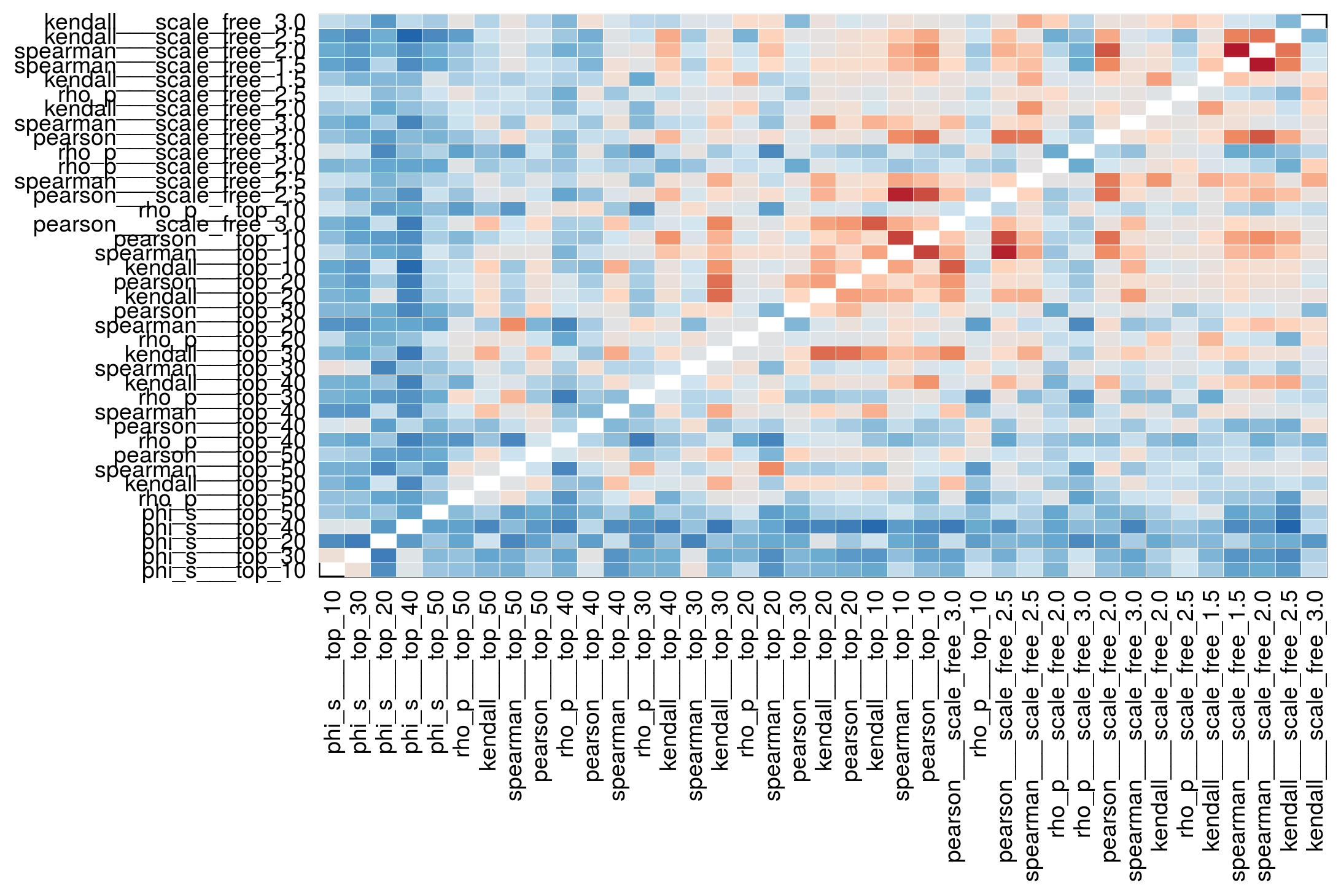

Supplement: Figure 1 — Comparison of different methods to extract the gene network from smooth muscle cell coomunity in bladder. [file Image_1.png]

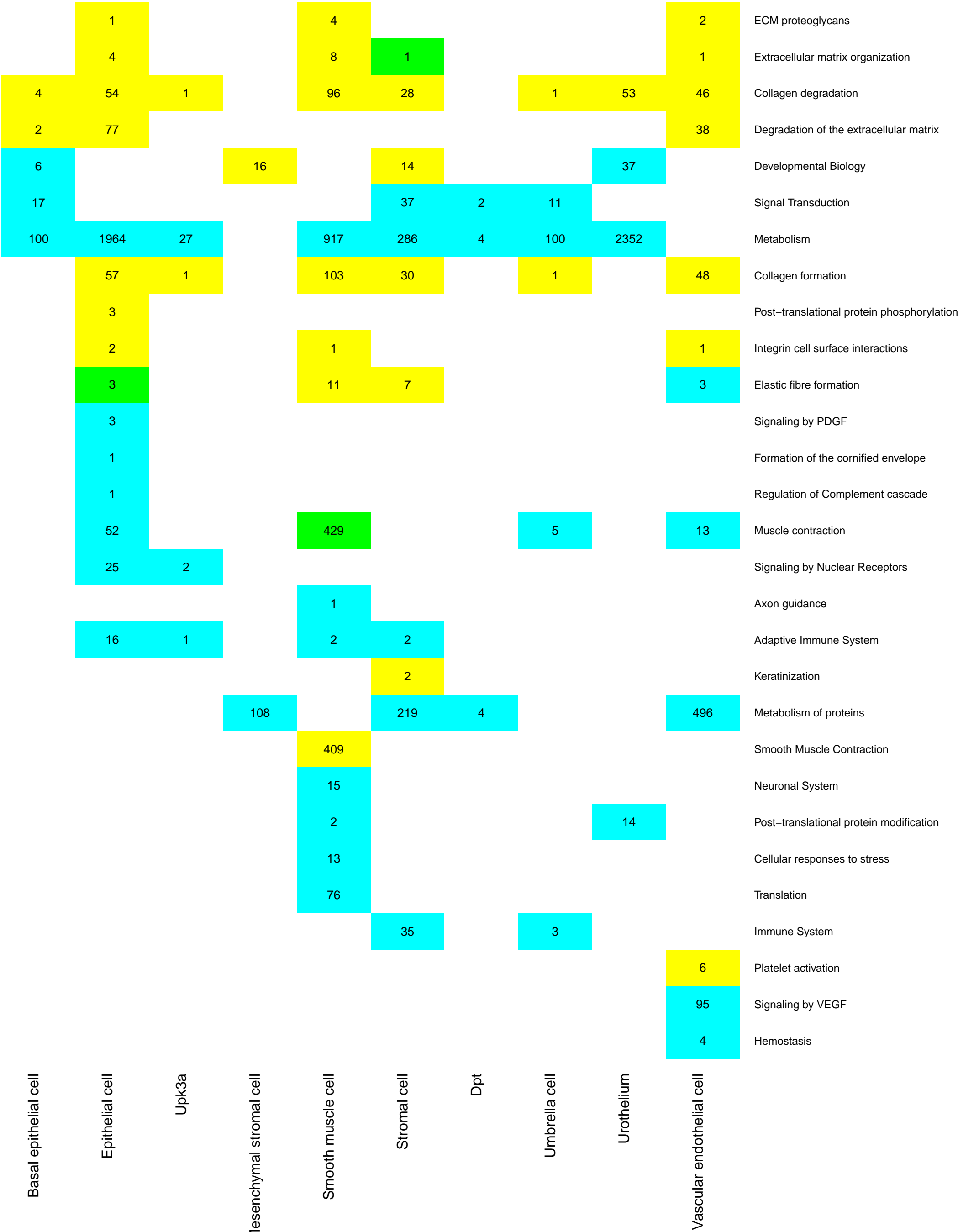

Supplement: Figure 2 — Rismed search on the Pubmed repository for bladder terms. [file DataSheet_1.pdf]
