# Supplementary material for: MTGO-SC, A Tool to Explore Gene Modules in Single-Cell RNA Sequencing Data
Source: Front Genet. 2019 Oct 9;10:953. doi: 10.3389/fgene.2019.00953 (PMC6794379; doi:10.3389/fgene.2019.00953)
Supplement: Figure 3 — Rismed search on the Pubmed repository for peripherl blood terms. [file DataSheet_2.pdf]

	B−cell	Ly6d	Erythroblast	Macrophage Ace high	Macrophage	Ace	S100a4	Monocyte	Elane	Natural Killer cell	Gzma	Neutrophil Camp high	Neutrophil	Camp	Neutrophil Il1b high	Il1b	T−cell
MHC class II antigen presentation	508																2641
Signal Transduction								7035	3	2382	15	14	5058	1521			19714
Metabolism	39025	18		17	54415	5417		49813	43	18596	140	41	49686	5214	36	1072	133436
Post−translational protein phosphorylation								136					70	34			246
Platelet degranulation													130	5			
Cell surface interactions at the vascular wall	2							12					12				
Adaptive Immune System								1846				1	1526	59			
Neutrophil degranulation	23				196	2		124	2	18	1	2	1370	17	1	2	57
Metabolism of proteins			803														121098
Post−translational protein modification								475	4								
Translation												2	3903	723			
Immune System					54954		51										
Toll−like Receptor Cascades					31												
Platelet activation			10														629
Hemostasis					792			1483									453
Antimicrobial peptides	3501	1	55	1	7784	134	10	6287	4	4944	25		5217		5	165	29889
Cell Cycle, Mitotic	65																96
Metal sequestration by antimicrobial proteins					22			3		1							4
Cell Cycle	3423				1390		7										
M Phase	8628				7314		11										
Cell Cycle Checkpoints	105				56												
Signaling by Rho GTPases	90																
Signaling by Interleukins	1304												1453		18	264	7276
S Phase	197																
Detoxification of Reactive Oxygen Species	4		1										33	1			12
Metabolism of porphyrins			27														
Heme biosynthesis			46														
Metabolism of RNA			167														
Metabolism of nucleotides			39														
Synthesis of DNA			142										2688		2	69	
Metabolism of lipids			31		11234		6										
Cytokine Signaling in Immune system				1	6143	22											16414
Mitotic Spindle Checkpoint					3												20
Oxidative Stress Induced Senescence					48												
PD−1 signaling								60									814
TCR signaling								59									4634
Peptide hormone metabolism										309	1						
Translation initiation complex formation													12	2			
Innate Immune System													5744		5	118	
Activation of Matrix Metalloproteinases																	271
DAP12 signaling																	51
Membrane Trafficking																	568
